# Does Perceived Lack of Control Lead to Conspiracy Theory Beliefs? Findings from an online MTurk sample

**DOI:** 10.1371/journal.pone.0237771

**Published:** 2020-08-17

**Authors:** Ana Stojanov, Jesse M. Bering, Jamin Halberstadt

**Affiliations:** 1 Department of Psychology, University of Otago, Dunedin, New Zealand; 2 Centre for Science Communication, University of Otago, Dunedin, New Zealand; Institut VEDECOM, FRANCE

## Abstract

It is widely believed that conspiracy theory beliefs are the product of perceived lack of control. However, to date there is mixed evidence, at best, to support this claim. We consider the reasons why conspiracy theory beliefs do not appear to be based in any straightforward way on control beliefs, interrogating existing findings and presenting new data that call the relationship into question. Across six studies conducted online using MTurk samples, we observed no effect of control manipulations on conspiracy theory beliefs, while replicating previously reported correlational evidence of their association. The results suggest that conspiracy beliefs are not suitable for compensating for threats to control. We discuss possible reasons for the discrepancy between experimental and correlational effects and examine the limitations of the studies.

## Introduction

*The world is ruled by a secret cabal*. *The US government was involved in the 9/11 attacks*. *HIV is a man-made virus*. Such statements, which are promoted on the internet and elsewhere, are examples of conspiracy theories: implausible, unwarranted claims that important social events are caused by malevolent clandestine groups, that usually run in contradiction to the explanations offered by the relevant epistemic authorities, and that are embedded in a more general worldview [1].

Although researchers have tried to understand conspiracy theory beliefs–which to nonbelievers appear unwarranted by evidence, if not entirely irrational–from many perspectives, including personality theory [2, 3], intergroup identity [4–6], and cognitive bias [7–9], arguably the most frequent accounts rely on some notion of “control.” The claim that perceived lack of control is the natural breeding ground for conspiracy theory beliefs is a recurring theme in popular science (e.g. [10–14]), and many academics agree (e.g., [15–18]).

However, on closer inspection, neither the theoretical role of control in conspiratorial thinking, nor the empirical relationship between the two constructs, is entirely convincing. Although specific accounts differ in their emphasis, control accounts of conspiracy theories generally assume that the perceived loss of personal control is threatening to one’s need for order (e.g., [19]) or one’s schemas about the world (e.g., [20]). Conspiracy beliefs help compensate for these threats by ascribing control to external entities or organizations (e.g. [21–23]), or by creating new meaning frameworks via novel connections among unrelated events. For example, perceiving a connection between a tornado and the unusually long contrails observed the previous day, provides a basis for believing that the government (or some other entity) has deliberately sprayed chemicals in the sky to cause the tornado.

However, it seems equally plausible that conspiracy theories would have the opposite effect on the perception of control and order. A common theme among conspiracy theories is that reality is not what it seems; they are counter-normative narratives that suggest institutions and explanations that we take for granted are illusions–a revelation that, if taken seriously, seems bound to challenge the larger meaning-making systems individuals have come to rely on. Furthermore, although people desire order, they seek to optimize, not to maximize it, and it could be argued that conspiracy theories provide “too much” order, structure, and meaning by accounting for errant data, connecting seemingly unconnected events and leaving nothing unexplained [24, 25]. Finally, even if conspiracy theories represent an effective means of restoring order and structure, they are not the only means [19, 26–28], and, it would seem, a means of last resort. While control accounts do not require that sources of control or meaning be benevolent, people theoretically favour control systems that are both “culturally accessible” and “socially acceptable” [27, 29]. In most—if not all—conspiracy theories, however, the alleged conspirators are malevolent [30], thus making them poor candidates for compensatory processes when numerous effective and socially sanctioned alternatives exist.

The experimental evidence for conspiracies as compensatory sources of personal control is as ambivalent as its theoretical rationale. In a typical experiment, researchers ask participants to write about a time they did not have control, and to compare these participants’ conspiracy beliefs with a control group that is asked to describe an event when they felt in complete control (or about a neutral event). Some studies report stronger conspiracy beliefs in the low/neutral control group compared to the high control group (e.g. [31–33]), but others report weaker ones (e.g. [34]), and yet others (e.g. [35, 36]) report no effects of control on conspiracy beliefs at all.

One factor in the variability of results is the corresponding variability–and sometimes dubious validity–of how belief in conspiracy theories is operationalized and measured. For example, Whitson and Galinsky [33, Study 4] found that participants recalling uncontrollable situations were more likely to perceive patterns in ambiguous stimuli (e.g., images in visual noise) and links between events (e.g., an increase in e-mail correspondence and a lack of a promotion). However, while conspiracy theories are related to illusory pattern perception [37], they are not reducible to it. And while novel connections between seemingly unrelated events can, of course, be the building blocks of conspiracy theories, such theories tell a more complex story that taps into a broader worldview. For example, seeing a connection between the “umbrella man” (a man who opened and lifted an umbrella when Kennedy’s limousine approached) and the assassination of Kennedy is a starting point for building a conspiracy theory, but it is not a conspiracy theory per se (e.g., that Kennedy’s assignation was an organized Soviet-backed plot carried out for political purposes).

In another operationalization of conspiracy belief, Van Prooijen and Acker [32] asked participants about the construction of the North-South metro line in Amsterdam, by posing situations that were technically conspiracies, but also plausible abuses of power warranting legitimate skepticism. Accusations such as, “The city council transferred parts of the budget to the bank accounts of others”, and “Members of the city council received money from construction companies to set this plan in motion,” describe political corruption, which differ from “conspiracy theories” in scope, plausibility, official endorsement, and psychological interest. Indeed, recent evidence indicates that belief in conspiracy theories and belief in corruption are two distinct psychological constructs, predicted by different aspects of a larger construct (“conspiracy mentality”; [1]).

Even studies that explicitly ask about beliefs in specific conspiracy theories may not be well suited for assessing the construct of conspiracy theory belief. For one, participants can easily recognize such items as “conspiracy theories,” whose negative connotations are likely to produce socially desirable responses [1]. Moreover, using specific conspiracy beliefs limits the generalizability of the findings to very particular and culturally specific claims, and may say little about a person’s general tendency to employ conspiratorial logic. More relevant, we argue, is an individual’s tendency to engage in conspiracy thinking, rather than their belief in any particular claim.

Given the significance and potential consequences of widespread conspiracy beliefs [38, 39], and the plausible but largely unsubstantiated role of control in their appeal, we here report three studies to test the effects of lack of control on conspiracy theory beliefs using a standard priming paradigm and a validated measure of *conspiracy ideation*, which reflects the belief that a powerful entity lies behind significant social or political events and that the conventional (official) truth is not the “real” truth. A fourth study confirms the results with a measure of specific claims, and two final studies examine two of the most likely moderators of control effects: the degree of control that a particular conspiracy claim affords; and the effectiveness of alternative means of restoring control. Although we conceptually replicate previous correlational work–lower control beliefs were associated with stronger conspiracy ideation–we find no evidence for a causal effect.

## Study 1

### Method

This and subsequent studies were approved by the University of Otago Human Ethics Committee. Before the beginning of the study participants read an information page and gave written consent for participation; after the study they were debriefed online.

#### Participants

In this and subsequent studies, sample size was determined a priori. Detecting an effect of *f* = 0.2, the average effect size reported in psychology research [40] with alpha = 0.05 and power of 80%, required 246 participants. Expecting some attrition, we recruited 301 Mechanical Turk (MTurk) workers (105 male, 195 female, 1 other), who completed an online questionnaire designed and presented in the Qualtrics survey environment. The mean age of the sample was *M* = 36.20 (range 18–79 years, *SD* = 12.24). The majority had Bachelor’s degree (41.2%), while 27.9% had a high school degree, 16.6% Master’s degree, 3% Doctoral degree, 2.7% no degree; 8.6% reported their education level as “other”.

#### Materials and procedure

Participants first read a general information sheet, which explained the tasks that they would be completing, but did not reveal the hypotheses being tested; the passive deception was revealed and explained in post-experimental debriefing. The experiment was described as a study of memory, in which, participants would be asked to recall and describe an event twice, once at the beginning of the study and once “after a break interval,” during which they completed the key dependent variables. Following Whitson and Galinsky [31], we used the to-be-recalled event as our manipulation of control. The low control group described an event in which they “did not have any control over a situation,” while the high control group described an event in which they “were in complete control of the situation.” Both groups were instructed to include “what happened, how you felt, etc.,” and to write at least six lines of text. A neutral group was asked to recall and write about their dinner the previous night. The wording of the low and high control manipulation was taken verbatim from Whitson and Galinsky [31] with the exception of the instruction for the length of the text.

To assess the effectiveness of the manipulation we measured participants’ feelings of control on Pearlin and Schooler’s [41] Mastery Scale. It consists of seven items (e.g., *I have little control over the things that happen to me*) that capture the extent to which people see themselves as being in control over their life [42], and which has been used in numerous studies as a measure of perceived control [43, 44]. The scale was anchored at 1 (strongly disagree) and 4 (strongly agree). Five items were reverse scored, such that higher scores indicate higher levels of control. In the current study Cronbach’s alpha was 0.84.

Conspiracy beliefs were measured on the conspiracy theory ideation subscale of Stojanov and Halberstadt’s [1] Conspiracy Mentality Scale. The subscale consists of seven items capturing the abstract premises of conspiratorial thinking, such as the belief in a powerful entity that controls significant social or political events. (A second dimension of the scale, measuring rational scepticism, is not relevant to the current study.) The subscale has good psychometric characteristics (in the current sample Cronbach’s alpha was 0.91) and convergent, divergent and predictive validity, and its construct validity has been confirmed in United States, New Zealand and North Macedonia populations [1]. Participants rated their agreement with each statement on a 1-to-7 scale anchored at “strongly disagree” and “strongly agree.” Each subscale was presented in a separate block, always in the same order (with the dimension of interest, conspiracy theory ideation, presented first). An attention check item (“To make sure you read attentively please select strongly agree”), was included in the second block.

After completing the dependent measure, participants were asked, in line with the cover story, to describe their event a second time (memory was not analyzed). Finally, they were asked to speculate about the study’s hypotheses, to answer basic demographic questions, and to assess the quality of their data (“Did you honestly answer the question in this survey? You’ll be paid regardless of how you answer”), before being debriefed.

### Results and discussion

We report all measures, manipulations, and exclusions in this and subsequent studies.

Twenty-nine participants were excluded from the analysis: sixteen because they failed the attention check question (low control = 5, neutral = 4, high control = 7); two because they assessed their data as unreliable (high control = 2) and 11 (low control = 7, high control = 4) because they expressed suspicion about the research aims (In studies 1–5, two research assistants coded whether the participants were naïve to the research aims. The inter-rater reliability ranged from 0.8 to 0.97, and disagreements were settled by the first author) leaving 272 participants in total (Including such participants does not change the pattern of results in any of the studies reported in this paper). A sensitivity analysis indicated that the study was able to detect a minimum effect size of *f* = 0.18.

The effectiveness of the control manipulation was tested with a one-way ANOVA on mastery scores, which revealed a significant effect of experimental condition *F* (2, 269) = 7.69, *p* < 0.001, *f = 0*.*23*. Post hoc Bonferroni comparisons indicated that the low control group (*M* = 2.64, *SD* = 0.50, *N* = 79) differed from both the neutral (*M* = 2.96, *SD* = 0.58, *p* < 0.001, *N =* 108) and the high control group (*M* = 2.89, *SD* = 0.58, *p* = 0.02, *N* = 85), but the high control group did not differ from the neutral group (*p* = 1.0). This is in line with other unsuccessful attempts to induce higher feelings of control compared to baseline levels [45, 46].

Contrary to the primary hypothesis, an ANOVA on conspiracy ideation indicated that the low control group (*M =* 3.66, *SD* = 1.17) had descriptively *lower* conspiracy beliefs than both the neutral (*M* = 3.99, *SD* = 1.27) and high control groups (*M* = 3.84, *SD* = 1.35), although the effect was not significant, *F* (2, 269) = 1.55, *p* = 0.215, *f* = 0.11. Excluding the high control group (in which the manipulation was ineffective), revealed a marginally significant difference *t* (185) = -1.815, *p* = 0.07, *d* = 0.27; the low and high groups did not differ, *t* (162) = - 0.925, *p* = 0.36, *d* = 0.14.

Finally, we calculated the Pearson’s correlation coefficient between conspiracy beliefs and the mastery score, finding a significant negative relationship, *r* = -0.237, *p* < 0.0001.

In sum, despite using a standard and empirically successful manipulation of personal control [31, 47, 48], we did not find that those whose control was threatened expressed greater conspiracy ideation; if anything, the tendency was for the opposite to be true. However, weaker feelings of control (independent of experimental condition) predicted stronger conspiracy ideation, as in previous research [32, 34, 49].

In Study 2, we attempted to replicate the results of Study 1 with a conceptually similar but arguably stronger manipulation of control.

## Study 2

### Method

#### Participants

Three hundred MTurk participants (137 male, 163 female) completed a questionnaire in the online survey platform Qualtrics. The mean age of the sample was 37.28 years (*SD* = 11.66, age range 18–71 years). The majority had an undergraduate degree (45%), a third (30.3%) had a high school degree, 14.4% had a graduate degree, 9.7% other and 0.7% no degree. None of the participants had taken part in Study 1.

#### Procedure and materials

After providing informed consent, participants were randomly assigned to one of three groups. Those in the high control group were informed that we were interested in the types of situations that people experience as controllable (or in the low control group, as uncontrollable). For additional clarity, the instructions defined the construct of control, noting that “a person is ‘in control’ of something to the extent that they are able to direct or influence it if they want to.” Participants were asked to take a moment to think about some of the situations in their life where they have control (or no control), and to briefly describe ten such situations in a separate box. After providing ten examples they were administered the Mastery Scale as a manipulation check (see Study 1). The neutral group proceeded immediately to this scale without engaging in any recall activity. As in Study 1, after completing the dependent measures, participants were invited to speculate about the experimental hypothesis before providing demographics and assessing their data quality.

### Results and discussion

Twenty-nine participants were excluded from analysis: nine (low control = 2, neutral = 2, high control = 3) because they failed the attention check question, one (low control) because they self-assessed their data as unreliable and 19 (low control = 4, high control = 14, neutral = 1) because they were not naïve to the research aims. A sensitivity analysis indicated that the study was able to detect a minimum effect size of *f* = 0.19.

Next, we checked the effectiveness of the manipulation. A one-way ANOVA revealed a marginally significant effect, *F* (2,268) = 2.610, *p* = 0.075, *f* = 0.14. A post hoc Bonferroni test showed that the low control group (*M* = 2.67, *SD* = 0.54, *N* = 72) differed marginally (*p* = 0.086) from the high control group (*M* = 2.87, *SD* = 0.55, *N* = 83) group, but that neither of these differed from the neutral (*M* = 2.82, *SD* = 0.56, *N* = 116). Testing the primary hypothesis, an ANOVA did not indicate a difference in conspiracy beliefs between condition (low control M = 3.96, SD = 1.12; neutral M = 3.77, SD = 1.29; high control M = 3.94, SD = 1.32), *F*(2,268) = 0.707, *p* = 0.494, *f* = 0.08. As in Study 1, there was significant negative correlation between the mastery and conspiracy beliefs scores, *r* = - 0.217, *p* < 0.001.

In sum, despite successfully (albeit weakly) manipulating control and using a conceptually strong measure of conspiracy thinking, neither of two well-powered studies detected any causal evidence for the compensatory hypothesis, though both revealed a correlational relationship.

It is worth considering, however, whether the manipulation check itself might be contributing to the null findings, by providing an opportunity to restore control, and thereby eschewing participants’ need for further efforts in the form of conspiracy belief change. Hauser, Ellsworth and Gonzalez [50] argue that manipulation checks as simple as asking participants about their feelings of control may serves the purpose of restoring their control. We note that, if this were true, it supports our contention that there are far easier ways to restore control than to endorse extreme, counter-normative belief systems. Nevertheless, we considered this possibility in Study 3. If the presence of the self-report measure of control was responsible for the null effect of the control manipulation on conspiracy ideation, then removing the manipulation check should produce the hypothesized relationship.

## Study 3

### Method

#### Participants

Two hundred and two MTurk workers (79 males, 123 females) participated. Half (51.5%) had undergraduate degree, a third (28.7%) high school degree, an eighth (12.9%) had a graduate degree, while 5.9% selected “other” as their highest level of education, and 1% reported they did not have a degree. The mean age of the sample was 39.14 years (*SD* = 12.62, range 20–71 years). No participants had taken part in previous studies reported in this paper.

#### Procedure and materials

The procedure was identical to Study 1, except that the manipulation check was omitted from the procedure, and that control was only manipulated downward.

### Results and discussion

Seven participants were excluded from the analysis: one (neutral group) because they self-assessed their data as unreliable, four (neutral = 3, low control = 1) because they failed the attention check question and two (both low control group) because they were not naïve to the research aims. A sensitivity analysis, indicated that the study is able to detect a minimum effect size of *f = 0*.*2*.

The low control group scored numerically higher on the conspiracy theory ideation (*M* = 3.33, *SD* = 0.96, *N* = 86) than the neutral group (*M* = 3.19, *SD* = 1.09, *N* = 109), but the difference was not significant, *t (*193) = 0.936, *p* = 0.350, *f* = 0.06. Thus, it does not appear that the inclusion of the manipulation check interfered with participants’ hypothesized motivation to restore control via conspiratorial thinking.

Studies 1–3, using a novel (but, we argue, more theoretically appropriate) operationalization of conspiracy beliefs, revealed no empirical support for the control hypothesis. To explore whether similar results would be obtained with a more typical operationalization of conspiracy beliefs (i.e. as specific claims), and therefore exclude the possibility that the null effects are unique to the conspiracy ideation scale, we replicated the studies again, this time using the Beliefs in Conspiracy Theories Inventory [3].

## Study 4

### Method

#### Participants

Two hundred MTurk workers (72 male, 126 female, 2 other) participated. A third had an undergraduate degree (39.5%), a third a high school degree (29.5%), while 19.5% had a graduate degree, 1.5% did not have a degree, and 9% had an “other” degree. The mean age of the sample was 40.85 years (*SD* = 13.44, range 20–82 years). No participant had taken part in the previous studies reported here.

#### Procedure and materials

The procedure was identical to Study 3 with the exception of the dependent measure. Instead of conspiracy ideation, we measured belief in fifteen specific conspiracies (e.g., *A powerful and secretive group*, *known as the New World Order*, *are planning to eventually rule the world through an autonomous world government*, *which would replace sovereign government)*, using the Belief in Conspiracy Theories Inventory (BCTI), developed and later modified by Swami and colleagues [3, 51]. (We used the modified version) Participants rated the conspiracy theories on a 1 (completely false) to 9 (completely true) scale, with higher scores indicating higher conspiracy theory beliefs.

### Results and discussion

Eight participants (all low control group) were excluded from the analysis, one because they self-assessed their data as unreliable and seven because they were not naïve to the research aims. A sensitivity analysis indicated that the study was able to detect a minimum effect size of *f = 0*.*20*.

The neutral group scored numerically higher on conspiracy theory beliefs (*M* = 3.31, *SD* = 1.57, *N* = 109) than the low control group (*M* = 3.27, *SD* = 1.65, *N* = 83), but the difference was not significant *t* (190) = 0.157, *p* = 0.88, *f* = 0.01. Ostensibly, then, the null effects in Studies 1–3 do not appear to be due to the instrument used to measure conspiracy theory beliefs.

Although our four studies show little evidence for a main effect of control priming on conspiracy beliefs, it is entirely possible that additional factors moderate the effects of control—that control motivates conspiracy beliefs in some circumstances but not others. In the final two studies, we explore two of the most plausible possibilities: the degree to which a conspiracy theory itself implies a controlling entity (Study 5); and the availability of other means of restoring control when it is threatened (Study 6).

## Study 5

If people turn to conspiracy theories as a compensatory control mechanism, it may be that some theories are more effective at restoring perceptions of order than others. In particular, threats to control may only prompt belief in conspiracies involving a controlling entity (e.g., the theory that the U.S.S.R. started the “hippie” movement in the United States in order to undermine and weaken America’s traditional values), because the belief that *someone* is in control is sufficient to satisfy the higher order need for structure and meaning. Other theories in which a controlling entity is absent or only implicit (e.g., the theory that Osama bin Laden died prior to the 9/11 terrorist attacks but was used as a scapegoat) may not restore control, or do so to a lesser extent. Consistent with this proposal, Kay et al. [27] found that participants whose control was threatened increased their belief in God, but only when God was described as controlling, rather than as a creator. Thus, in Study 5, we developed a new stimulus set to test the hypothesis that participants whose control was threatened would be inclined to vest more belief in specific conspiracy theories about controlling entities, but not in other conspiracy theories.

### Method

#### Participants

The participants were three hundred MTurk workers (108 males, 192 females, *M* = 37.52, *SD* = 12.001). The majority (44%) had an undergraduate degree, while 29% had a high school degree, 13% had a master’s degree, 2% a doctoral degree and 1% did not have a degree. The mean age was 37.54 years (range 18 to 73 years). None had taken part in our previous studies.

#### Materials

The dependent measure was developed in several stages. First, an independent group of MTurk participants (*N* = 210) were asked to write five conspiracy theories that they had heard before, from which we identified 112 unique exemplars. Next, two research assistants were asked to code these theories on a 9-point scale anchored at 1 (“the goal of this conspiracy is NOT control”) and 9 (“the goal of this conspiracy is control”). The intraclass correlation coefficient was *r* = 0.84. The final rating comprised the average of the ratings of the two research assistants. Next, a second group of 85 MTurk participants was asked to rate the conspiracy theories in terms of their prototypicality, using a seven point scale anchored at 1 (“a bad fit” with their idea of a conspiracy theory) and 9 (an “excellent fit” with their idea of a conspiracy theory). A third group of 96 MTurk participants was asked to rate the same conspiracies for likely veracity, using a nine point scale anchored at 1 (“definitely false”) and 9 (”definitely true”). The intraclass correlation coefficient for prototypicality ratings was *r* = 0.89 and for the veracity *r* = 0.96. The prototypicality and veracity scores for each conspiracy theory were obtained by averaging ratings, respectively across all participants.

These extensive pretest data were used to create seven pairs of conspiracy theories that were equated on veracity (*t* (95) = 0.604, *p* = 0.55, *d* = 0.06) and prototypicality (*t* (84) = 0.439, *p* = 0.66, *d* = 0.05), but that each differed by at least 5 scale points in terms of control (*Ms* = 8.21 vs. 1.71). For the full list of the selected conspiracies, along with their control, prototypicality and veracity rating (obtained from the pre-test) see Table 1, for the full list of all pretest conspiracies see the online supplementary materials.

**Table 1:** Conspiracy theory pairs.

	Low control theory	C	V	P	*M* (*SD*) low	*M* (*SD*) neutral	*M* (*SD*) high	High control theory	C	V	P	*M* (*SD*) low	*M* (*SD*) neutral	*M* (*SD*) high
1	Lyme disease is a cross between aids and syphilis that escaped the lab via an infected tick.	1.50	1.92 (1.83)	4.69 (2.98)	1.95 (1.57)	1.73 (1.21)	1.72 (1.24)	Soy is being used to feminize men.	7	1.97 (1.87)	4.74 (2.99)	1.79 (1.35)	1.70 (1.19)	2.00 (1.43)
2	There is a secret alien base on Antarctica	1	2.21 (2.04)	4.89 (3.24)	1.89 (1.54)	2.03 (1.49)	2.22 (1.70)	The USSR only appeared to collapse to cause a false sense of safety in the West.	6.50	2.32 (1.97)	4.92 (2.83)	2.05 (1.28)	2.12 (1.34)	2.14 (1.27)
3	Bigfoot, a human-animal hybrid, is a scientific experiment gone wrong.	1.50	1.73 (1.37)	4.97 (2.94)	1.90 (1.45)	1.86 (1.33)	1.95 (1.42)	The weather is controlled by multiple governments to help various industries and hurt others.	9	1.86 (1.80)	4.99 (3.12)	1.78 (1.46)	1.72 (1.23)	1.85 (1.62)
4	There are aliens walking among us right now	1.50	2.81 (2.49)	5.07 (3.03)	2.34 (1.72)	2.39 (1.64)	2.61 (1.87)	The government planted cocaine in black neighbourhoods to keep black society addicted and helpless.	9	3.27 (2.71)	5.11 (3.13)	2.48 (1.88)	2.54 (1.82)	2.66 (2.02)
5	Russia sent numerous humans into space before Yuri Gagarin, but they died there.	2	2.61 (1.94)	5.18 (2.63)	2.54 (1.73)	2.80 (1.76)	2.50 (1.53)	Russia started the hippie movement in the US because it wanted to undermine and weaken America’s strong traditional values.	7.50	2.23 (2.23)	5.18 (3.08)	1.83 (1.33)	1.84 (1.33)	1.89 (1.30)
6	President Obama is secretly a Muslim who wasn’t born in Hawaii.	1.50	2.14 (2.33)	5.39 (3.14)	2.13 (1.84)	2.15 (1.79)	2.14 (1.80)	Jews secretly control the banking system and conspire for nefarious purposes.	9	2.21 (2.24)	5.44 (3.15)	1.92 (1.47)	1.74 (1.33)	2.15 (1.64)
7	Osama Bin Laden dies just before 9/11 but was used as a scapegoat.	3	2.27 (2.11)	5.28 (3.12)	1.92 (1.54)	1.92 (1.55)	1.89 (1.42)	The identification chips implanted into household pets are being used to spy on citizens.	9	2.26 (1.94)	5.58 (2.84)	1.84 (1.44)	1.90 (1.44)	2.14 (1.40)

C = average control rating during pre-test, V = mean veracity rating during pre-test (and its standard deviation), P = mean prototypicality rating during pre-test (and its standard deviation). Each pair is matched on prototypicality and veracity but differs on control rating. M (SD) = the mean rating and its standard deviation for the current study.

#### Procedure

The procedure was identical to that of Study 3, except for the use of two types of specific conspiracy theories, varying in the control they imply, and the addition of a high control group as additional comparison group. Participants rated their agreement with the 14 conspiracy statements, presented in a random order, on a 7-point scale (1 = strongly disagree, 7 = strongly agree). Mixed among these statements was an attention check question (i.e., “As a response to this question select strongly agree.”).

### Results and discussion

Nineteen participants were excluded from the analysis: nine (low control = 3, neutral = 6) participants due to failures of attention and ten (low control = 4, high control = 6) because they were not naïve to the research aims. A sensitivity analysis (α = 0.05, 1 - β = 0.8, correlation among repeated measures = 0.8) indicated that the study is able to detect a minimum effect size of *f = 0*.*06*.

A mixed ANOVA with control manipulation (low control vs. neutral vs. high control) as between subject factor, and type of conspiracy theory (control related theory or non-control related theory) as a repeated measure, revealed no main effect of the manipulation *F* (2, 278) = 0.250, *p* = 0.779, *f* = 0.03, (low control: *M* = 2.03, *SD* = 1.11, *N* = 92; neutral: *M* = 2.03, *SD* = 0.98, *N* = 115; high control: *M* = 2.13, *SD* = 1.12, *N* = 74), but a significant effect of conspiracy theory type *F* (1, 278) = 7.40, *p* = 0.007, *f* = 0.16, with participants expressing stronger belief in conspiracy theories that did not involve control (control related CT: *M =* 2.00, *SD =* 1.09; control non related CT: *M* = 2.12, *SD* = 1.14). Crucially for our purposes, there was no conspiracy theory type x control manipulation interaction *F* (2, 278) = 1.144, *p* = 0.32, *f =* 0.08 (The pattern of results was unchanged when we analyzed the low control vs. neutral groups only). We also analysed each pair separately. The interaction was not significant for any individual pair.

In sum, we found no support for the hypothesis that control threat motivates belief in theories that hypothesize a controlling entity. In addition, participants rated the theories as more believable when they did *not* afford control, an unexpected result given that the two types of theories had been matched for believability in pretesting. To explore this discrepancy further, we compared our participants to the pretest sample on key demographic variables: age, gender, education and political ideology. The analyses indicated a marginal difference in the gender distribution, *χ*^*2*^ *=* 4.98 (2), *p* = 0.08; there were a greater proportion of women in the main study compared to the pretest. In addition, women rated the control unrelated conspiracy theories as more believable than the control related, *t*(159) = -2.77, *p*<0.01, *d* = 0.21. When we re-ran the repeated measures ANOVA, with gender as a covariate, the main effect of conspiracy theory type was not present, *F* (1, 277) = 0.045, *p* = 0.83, *f* = 0.01 and neither were the main effect of group, *F* (2, 277) = 0.225, *p* = 0.8, *f* = 0.04, nor the interaction effect *F* (2, 277) = 1.15, *p* = 0.318, *f* = 0.09. Thus, the significant effect of conspiracy theory type was most likely due to the higher proportion of women in the main study.

## Study 6

Another plausible moderating factor determining the value of conspiracy theories for restoring threatened control, is the availability of *alternative* means of control. As noted above, while compensatory control processes do not require that sources of control be benevolent, people unsurprisingly favour sources of control that are not themselves threatening in other ways. Furthermore, there is a plethora of alternative control and meaning-making systems that are proven substitutes for personal control, such as God [52], government institutions [48], scientific [53], and even more abstract beliefs such as in meritocracy [54] and societal progress [55, 56]. Even if these alternatives are not all equally effective at establishing a sense of control, conspiracy theories would seem to be an option of last resort, given the essentially malevolent worldview they imply, and the social stigma that can accompany their endorsement [57]. Consequently, conspiracy theorizing may be a secondary strategy for restoring control, avoided when one has other means of affirming that the world is ordered [58].

Thus, in Study 6, we tested the hypothesis that threats to control would increase belief in conspiracy theories to a greater degree (or only) when the effectiveness of a second salient source of control–the government [29]–was called into question.

### Method

#### Participants

Six hundred and five MTurk volunteers participated (267 male, 337 female, 1 “other”). The majority (42.6%) had an undergraduate degree, 24.5% had a high school degree, while 21.3% had a graduate degree, 9.3% an “other” degree and 2.3% had no degree. The mean age was 33.96 years (*SD* = 11.11 age range 18–78). None had participated in our previous studies.

#### Materials and procedure

Participants were randomly assigned to a low control or a neutral group, as described in previous studies, and, independently, to a “competent government” or “incompetent government” condition. In the former, participants read a passage (about which they expected to answer questions) describing the U.S. government’s response, generally considered competent and effective [59], to Hurricane Irma which most severely struck Florida, Georgia and South Carolina in 2017; in the latter, they read a similar passage about the government’s response to Hurricane Katrina, generally considered incompetent and ineffective [60], which struck Florida and Louisiana in August 2005. For example, Hurricane Irma’s response was described as “timely, well managed, planned and coordinated. Residents were ordered to a shelter of last resort with sufficient provision of food, water, security and sanitary conditions. No one was thirsty, exhausted or victim to violence.” Hurricane Katrina’s response was described as “delayed, mismanaged, unprepared and uncoordinated. Residents were ordered to a shelter of last resort without provision of adequate food, water, security or sanitary conditions. Several citizens died of thirst, exhaustion and violence, days after the storm had passed.” (The full text of both scenarios appear in the Appendix.)

Afterwards, consistent with the cover story, participants answered several multiple choice questions about the scenario (which were not analyzed). Afterwards, all participants completed the Conspiracy Mentality Scale, as described in Study 1, including an attention check, followed by demographic questions and self-assessment of data quality.

### Results and discussion

One hundred and four participants were excluded from the main analysis: 74 due to failures of attention and 30 due to failure to following instructions. This left a total of 501 participants. A sensitivity analysis (α = 0.05, 1 - β = 0.8) indicated that the study is able to detect a minimum effect size of *f = 0*.*12*.

A 2 (control priming: low vs. neutral) x 2 (government competence: competent versus incompetent) ANOVA on conspiracy theory ideation revealed no main effect of priming, *F* (1, 497) = 0.161, *p* = 0.689, *f* = 0.02 or of competence, *F* (1, 497) = 0.243, *p* = 0.623, *f* = 0.02. Importantly, there was no interaction *F* (1, 497) = 1.301, *p* = 0.255, *f* = 0.05, (see Table 2 for descriptive statistics). Thus, the study, again, provides no evidence that control priming influences conspiracy beliefs, regardless of whether an alternative source of control has been compromised.

**Table 2:** Descriptive statistics by recall task and scenario type.

Control priming	Scenario	Mean	SD	N
Low control	Incompetent	4.16	1.23	109
	Competent	3.98	1.39	125
Neutral	Incompetent	4.08	1.25	133
	Competent	4.15	1.07	134
Total	Incompetent	4.11	1.24	242
	Competent	4.06	1.23	259

## Meta-analyses

**Fig 1:**
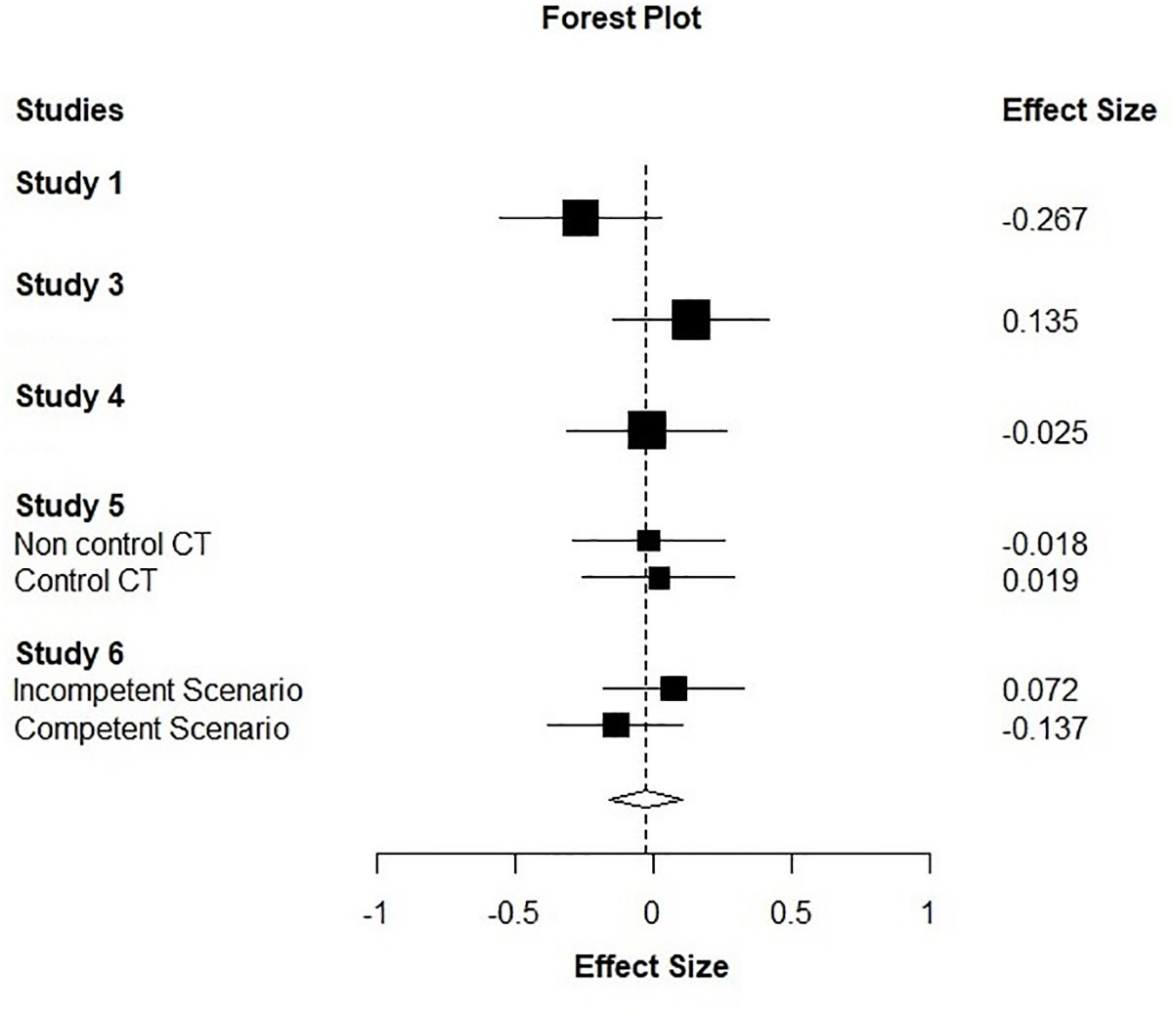
Forest plot of analysed effect sizes from Studies 1and 3–6 (squares) and the cumulative effect size (diamond).

Although Studies 1–6 varied somewhat in methodology, their consistent inclusion of low control and neutral conditions permitted a meta-analysis, which maximizes both power and accuracy of our effect size estimates. Because in Study 2 the low control manipulation did not lower perceived control compared to the neutral group we excluded Study 2 from the analysis. For Studies 5 (low control compared to neutral) and 6 we looked at the effect at each level of the moderator. Because the effect sizes in Studies 5 and 6 were not independent we used the robumeta [61] package in R, which takes the dependency into account, to conduct the meta-analysis. A test of heterogeneity suggested consistency between the studies, *I*^*2*^ = 11. The cumulative effect size did not suggest effect of condition, *d = -*0.03, 95% CI [-0.20, 0.13], *p* = 0.60 (Fig 1).

To investigate support for the null hypothesis relative to the alternative hypothesis that control priming influences conspiracy theory beliefs, we used a Bayesian information criterion (BIC), using the calculator by Masson [62], based on Wagenmakers [63]. The analysis requires transformation of the sum of squares generated by an ANOVA output and provides graded level of evidence for the null and alternative hypothesis. According to Raftery [64], the strength of evidence for the posterior probability of a hypothesis given the data (*p*_*BIC*_(H_1_|D) within the range of 0.50–0.75 is “weak”, between 0.75–0.95 is “positive”, between 0.95–0.99 “strong” and above 0.99 “very strong”.

For those studies containing three groups we analysed the low and neutral (Study 1 and 5) or low and high (Study 2). For studies 5 and 6 we looked at each level of the moderator.

Table 3. shows the posterior probability of the null hypothesis given the data, *p*_*BIC*_(H_0_|D), and the probability of the alternative hypothesis given the data, *p*_*BIC*_(H_1_|D). There is positive evidence for the null hypothesis in five out of the six studies. In Study 1, there is only weak evidence for the null hypothesis, but the support for the alternative hypothesis is even lower (unsurprisingly, given that the effect is in the “wrong” direction).

**Table 3:** Overview of study design and posterior probabilities for H_0_ and H_1_.

	Manipulation	Manipulation check	DV	*p*_*BIC*_(H_0_|D)	*p*_*BIC*_(H_1_|D)
Study 1	Recall task	✓	CTI	0.60	0.40
Study 2	Modified recall task	✓	CTI	0.93	0.07
Study 3	Recall task		CTI	0.90	0.10
Study 4	Recall task		BCTI	0.93	0.07
Study 5	Recall task (Control related)		Specific theories	0.93	0.07
Study 5	Recall task (Control non related)		Specific theories	0.93	0.07
Study 6	Recall task (Irma Scenario)		CTI	0.90	0.10
Study 6	Recall task (Katrina scenario)		CTI	0.93	0.07

CTI = The Conspiracy Theory Ideation Subscale from the Conspiracy Mentality Scale; BCTI = Beliefs in Conspiracy Theories Inventory

## General discussion

Six studies, conducted online and using MTurk participants, tested the hypothesis that lack of control motivates belief in conspiracies. While the hypothesis is plausible, there was in fact little support for it in the (small) literature on the subject. The current findings offer no further evidence, and some positive evidence for the null hypothesis, at least when the most common experimental manipulation of control is used with online samples. Participants who were asked to recall instances in which they felt out of control were no more likely to endorse either general conspiracy beliefs, or specific conspiracy theories, relative to participants who recalled neutral memories, or nothing at all. Plausible moderating variables–the extent to which conspiracies provide evidence of control (Study 5), and the effectiveness of an alternative means of control (Study 6)–had no effect, and a meta-analysis of the studies determined a cumulative effect size not statistically different from zero. On the contrary, a Bayesian analysis indicated that the null hypothesis (i.e., that there is no causal relationship between lack of control and conspiracy theory beliefs) should be retained.

How to explain the inconsistency between previous positive results and our null findings? One explanation, of course, is that previous “positive” findings were simply Type 1 errors, which is not farfetched given social psychology’s historical inattention to power [65, 66] combined with publication bias. Indeed, assuming the “actual” effect size of control threat on conspiracy beliefs is 0.2 (the average effect size reported in psychology research [67]), then previous studies reporting positive results may have included chance findings given their low power. For example, assuming the actual effect is 0.2, then Whitson and Galinsky’s [31] Study 1 has power of 0.17, Sullivan et al.’s [33] has power of 0.18, and van Prooijen and Acker’s [32] power of 0.22. By contrast, studies finding nonsignificant findings, such as those by Hart and Graether’s [35] or Nyhan & Zeitzoff [36], have higher power, 0.52 and 0.88, respectively. Another possibility, however, is that some previous studies measured constructs other than “pure” conspiracy ideation (e.g., pattern perception, paranoid beliefs, corruption beliefs), which *are* susceptible to control threats. Indeed, studies that examine more typical conspiracy theories and have larger samples tend to report null results. For example, Hart and Graether [35], found no effects of control on the Generic Conspiracist Beliefs Scale (another psychometrically validated measure of conspiracy beliefs, with items such as, “Experiments involving new drugs or technologies are routinely carried out on the public without their knowledge or consent”). Similarly, Nyhan and Zeitzoff [36] found no effect of control priming on Middle Eastern and North African participants’ conspiracy beliefs, using a list of conspiracy theories consisting of items such as “Jewish leaders are secretly conspiring to achieve world domination.”

It is also worth repeating that the current results were obtained entirely with online samples, while most studies in this area (including the majority of those with positive effects of control) have been conducted in the laboratory (typically with student samples). In principle, online manipulations of control could be weaker than those in the laboratory, accounting for the lack of support for the control hypothesis. As an empirical matter, however, there is no evidence that effects in this paradigm depend on sample type [68]. In a recent meta-analysis of experimental manipulations of control on conspiracy beliefs [68] conducted on 45 effect sizes across 23 studies (including those reported here), we found no moderating effect of sample type (MTurk vs. students vs. other). Thus, although it is important to continue to examine the role of control threats in diverse samples and contexts, the current data, despite being collected online, nevertheless challenge the hypothesis that such threats account for conspiracy beliefs in any significant way.

Although there was no evidence in the current studies for a *causal* effect of control on conspiracy beliefs, Studies 1 and 2 provided evidence for a *correlational* relationship. Such correlational findings are consistent with previous research [32, 34, 49, 69, 70], suggesting that chronic, rather than acute, feelings of low control are related to higher conspiracy theory beliefs. While longitudinal studies may help shed light on the effects of chronic lack of control on conspiracy beliefs, it is also possible that the link is spurious. There are any number of co-variates of both lack of control and conspiracy theory belief, including stress [71, 72], self-esteem [73, 74], anxiety [75, 76] or uncertainty [77], that could account for their association. Also, the correlation may be due to conspiracy beliefs’ shared variance with paranoia (see for example Imhoff & Lamberty, [78]). Alternatively, the causality may run in the opposite direction: conspiracy theories themselves may reduce feelings of control. Jolley and Douglas [79], for example, found that participants exposed to conspiracy theories about vaccines reported greater feelings of powerlessness compared to participants exposed to anti-conspiracy material, suggesting that conspiracy beliefs *threaten* rather than *satiate* one’s sense of control. Furthermore, it is not clear what type of correlational relationship should be taken as evidence for satiation; if conspiracy theories compensate for compromised personal control, should the relationship between control and conspiracy beliefs in cross-sectional studies be positive or negative? Thus, correlational evidence in the absence of experimental evidence is not a strong foundation for claiming that lack of control is a precursor to conspiracy beliefs.

Even if our present null results are confirmed in subsequent research, it is important to acknowledge their limitations. Priming is only one method of threatening (or bolstering) a sense of personal control, and while we found no evidence for changes in conspiracy beliefs, more potent or qualitatively distinct experimental manipulations could conceivably produce effects that we were unable to detect here. Furthermore, we could not assess the effects of chronic lack of control: It may be that prolonged periods of control deprivation do indeed produce reliance on other, even malevolent sources of control, including conspiracy theories.

Another unexplored area is whether lack of control in a specific domain will lead to belief in conspiracy theory in that domain. For example, people diagnosed with HIV or COVID-19 might be particularly inclined to believe in conspiracy theories involving those viruses, rather than conspiracy theories in general. Indeed, in a recent naturalistic study [80], we found a domain-specific link between threat to control and conspiracy beliefs. For example, after a series of tornados, those who experienced such natural disasters were more likely to endorse weather-related conspiracy beliefs than they were other types of conspiracy beliefs. Moreover, the strength of these endorsements subsided over time (i.e., three months after tornado season). Such findings seem to suggest that the link between perceived control and conspiracy beliefs is both nuanced and transitory, and that there is no “one size fits all” explanation for lack of control and conspiracy-theory endorsement in general.

Finally, we relied exclusively on laboratory manipulations of control (or lack thereof). While such empirical techniques are widely used in the literature, the effectiveness of the “recall task” in threatening participants’ sense of control has been questioned [34], and its effects were weak in our studies. “Real life” events, such as natural disasters, terrorist attacks, and pandemics, may produce stronger threats to personal control (and indeed, evidence suggests that traumatic events are followed by an increase in conspiracy discourse [81]). Findings from our own naturalistic study seems to confirm this. However, such naturalistic “manipulations” may confound perceptions of control with other variables, such as threats to identity, uncertainty about the future, and stress. Thus, any naturalistic observations need to be accompanied by rigorous experimental research to triangulate the mechanism(s) responsible for any changes in conspiracy beliefs. On their own, both empirical approaches are problematic, in that experimental manipulations of control may be too “weak” to evoke conspiracy-belief endorsements, and perceived loss of control under naturally occurring conditions are confounded with other variables.

## Supporting information

S1 Data (SAV)

S2 Data (SAV)

S3 Data (SAV)

S4 Data (SAV)

S5 Data (SAV)

S6 Data (SAV)

S1 Appendix (DOCX)
