# Supplementary material for: Does Perceived Lack of Control Lead to Conspiracy Theory Beliefs? Findings from an online MTurk sample
Source: PLoS One. 2020 Aug 17;15(8):e0237771. doi: 10.1371/journal.pone.0237771 (PMC7430734; doi:10.1371/journal.pone.0237771)
Supplement: S1 Appendix — (DOCX) [file pone.0237771.s007.docx]

Appendix

These scenarios were adapted from information available at Wikipedia and <https://www.cato.org/blog/hurricane-katrina-remembering-federal-failures>

**Hurricane Katrina scenario**

The government has come under intense criticism in recent years for its ability to carry out its key functions. Nowhere was this better exemplified than by its response to Hurricane Katrina.

The government response to hurricane Katrina was delayed, mismanaged, unprepared and uncoordinated.

Residents were ordered to a shelter of last resort without provision of adequate food, water, security or sanitary conditions. Several citizens died of thirst, exhaustion and violence, days after the storm had passed.

Indecision plagued government leaders in the deployment of supplies, in medical personnel decisions, and in other areas. In places that desperately needed help, it took days to deliver medical supplies. Even the grisly task of body recovery after Katrina was slow and confused. Bodies went uncollected for days.

Further, federal agencies were unfamiliar with their roles and responsibilities. There was general confusion over mission assignments, deployments, and command structure.

 Agencies could not communicate with each other due to equipment failures and a lack of system interoperability. The Federal Emergency Management Agency (FEMA) was not able to coordinate its efforts with other federal agencies.

To conclude, the response to hurricane Katrina nicely illustrates government inefficiency at all levels, and serves as a reminder of its chaotic functioning.

**Hurricane Irma Scenario**

The government has come under intense criticism in recent years for its ability to carry out its key functions, but in fact the government has shown remarkable competence when it is needed. Nowhere was this better exemplified than by its response to Hurricane Irma.

The government response to hurricane Irma was timely, well managed, planned and coordinated.

 Residents were ordered to a shelter of last resort with sufficient provision of food, water, security and sanitary conditions. No one was thirsty, exhausted or victim to violence.

 Government leaders were decisive in deployment of supplies, in medical personnel decisions, and in other areas. In places that desperately needed help, medical supplies were quickly delivered. The body recovery after Irma was fast and organized.

Further, federal agencies were familiar with their roles and responsibilities. Mission assignments, deployments, and command structure were clear.

System interoperability and effective equipment allowed agencies to communicate with each other. The Federal Emergency Management Agency (FEMA) effectively coordinated its efforts with other federal agencies.

To conclude, the government response to hurricane Irma nicely illustrates government efficiency at all levels, and serves as a reminded of its orderly functioning.
